# Induced Proximity Approach Enables the Recombinant Production of Polyphosphorylated Silk Proteins with Improved Adhesiveness

**DOI:** 10.1021/acs.biomac.5c01431

**Published:** 2025-11-25

**Authors:** Nea B. Möttönen, Ruxia Fan, Stefania Aspholm-Tsironi, Salla Keskitalo, Antti Tuhkala, Markku Varjosalo, A. Sesilja Aranko

**Affiliations:** † Department of Bioproducts and Biosystems, School of Chemical Engineering, 174277 Aalto University, 02150 Espoo, Finland; ‡ Institute of Biotechnology and Helsinki Institute of Life Science, 3835 University of Helsinki, 00014 Helsinki, Finland

## Abstract

Phosphorylation is considered to play a role in many of the functional properties of silk proteins, affecting their solubility, environmental adaptability, adhesion, and biocompatibility. However, investigating these effects has been hampered by the difficulty of isolating phosphorylated proteins from natural sources and the limitations of the current *in vitro* phosphorylation techniques. Here, we present a novel *in vivo* phosphorylation strategy for recombinant silk proteins in *Escherichia coli*, utilizing an engineered SpyCatcher/SpyTag system to induce proximity between the target protein and kinase. This scaffolding approach enhances kinase specificity and minimizes off-target effects, increasing the phosphorylation efficiency while preserving cell viability. We demonstrate the applicability of this system to both dragline and aggregate spider silks. Furthermore, we show that polyphosphorylation enhanced the adhesive properties of silk proteins. This modular and tunable strategy provides a powerful platform for producing polyphosphorylated fibrous proteins, offering broad implications for biomaterial design and functional protein engineering.

## Graphical abstract

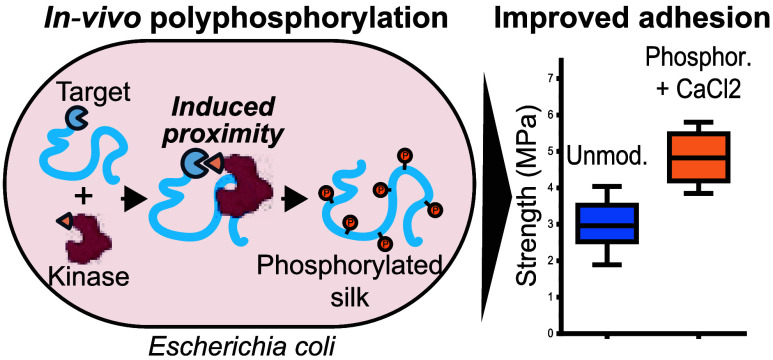

## Introduction

Post-translational modifications (PTMs) expand the functional capacity of the genetic code by increasing the proteomic diversity. Among these, phosphorylation is one of the most prevalent and functionally significant PTMs. Owing to its reversible nature, phosphorylation enables cells to dynamically regulate protein activity, localization, and interactions. It plays a critical role in cellular signaling pathways^1^ and is implicated in a wide range of diseases, including various cancers and neurodegenerative disorders.^1,2^

Beyond its crucial role in intracellular signaling, phosphorylations have been found in many secreted proteins.^3–8^ These include structural proteins, like silkworm^9^ and spider silks,^10–13^ as well as collagen.^14^ Secreted proteins with multiple phosphorylation sites are also found in body fluids, including caseins in milk^15^ and statherin in saliva,^16^ and are abundant in marine adhesives^8,17^ and biomineralization systems.^4^

Many of the secreted phosphorylated proteins do not fold into conventional globular structures and contain multiple or even dozens of phosphorylation sites. The accumulation of negatively charged phosphate groups can remarkably alter the physicochemical properties and interactions of phosphorylated proteins.^18,19^ The functional properties of phosphorylated secreted proteins are often mediated by interactions with calcium ions or hydroxyapatite, contributing to functions such as casein micelle formation in milk,^15^ underwater adhesion on caddisfly silk,^11^ and biomineralization.^4,20^

Phosphorylation is also prevalent in silk proteins, in which the modifications have been connected to physicochemical properties, as well as environmental adaptation of the silk fibers.^9,10,21^ Spider dragline silk, known for its exceptional tensile strength and toughness, and silkworm silk, widely utilized in the textile industry, both contain phosphorylations.^9,10,13^ While phosphorylations have been studied to affect the solubility of silk proteins and the ability to adapt to humid environments,^21,22^ it is yet unclear how they affect the mechanical properties and silk fiber formation.^23^ Phosphorylation of silk-based materials is thought to help in adhesion,^11,24^ enable self-healing of materials,^25^ as well as enhance the cytocompatibility of silk-derived materials.^26^ Especially, underwater adhesives have been reported to often contain post-translationally modified amino acids, especially phosphorylated serines and often hydroxylated tyrosines.^24^ Caddisfly silk, mussel byssus, and sandcastle worm adhesive exemplify protein-based sticky aquatic bioadhesives that enable organisms to adhere to underwater surfaces or bind materials such as rocks and sand for structural protection.^11,24,27,28^ In addition to underwater adhesives, aggregate silk forming the adhesive capture spiral in spider webs has also been shown to contain phosphorylations, which contribute to the glue-like properties of the silk.^21^

Despite its vast potential, studying the effects of phosphorylation on the properties of fibrous proteins remains challenging. One major obstacle is the difficulty in identifying post-translational modifications in fibrous proteins extracted from natural sources, largely due to the harsh chemical or mechanical treatments required for their isolation.^13^ These treatments can obscure or degrade phosphorylation sites, making it difficult to obtain phosphorylated proteins in a reliable or homogeneous form. To overcome these limitations, *in vitro* phosphorylation—either *via* chemical or enzymatic methods—has been employed on both recombinant proteins and proteins isolated from natural sources.^22,29–31^ While *in vitro* phosphorylation is suitable for proteins with accessible and stable phosphorylation sites, it presents several practical challenges. Enzymatic phosphorylation, for instance, requires time-consuming and costly production and purification of kinases.^32,33^ Additionally, *in vitro* methods often necessitate further purification steps^34^ and frequently result in proteins that are heterogeneously modified.^35^ Chemical phosphorylation methods also face significant hurdles, particularly the risk of unwanted side reactions. These become especially problematic when attempting to generate multiphosphorylated proteins, where controlling site specificity and reaction conditions is more difficult.

Coexpression of kinases enables *in vivo* phosphorylation, allowing modification of the target protein to occur immediately after translation. This strategy has been successfully applied, for instance, to the production of phosphorylated recombinant casein proteins.^36,37^ By incorporating phosphorylation into the expression process, this approach eliminates the need to produce and purify large quantities of kinases for *in vitro* use and avoids the additional processing step otherwise required to phosphorylate nonmodified proteins postexpression. However, the coexpression of kinases presents its own challenges. Kinase expression in recombinant systems can be problematic due to cytotoxic effects and the risk of off-target phosphorylation.^33^

To mitigate the cytotoxicity and off-target effects associated with modifying enzymes, increasing the specificity of these enzymes toward their intended target proteins is a promising strategy. One way to achieve this is by optimizing the kinase individually for each target protein. However, this approach is labor-intensive and impractical for large-scale or multitarget applications, as it requires extensive engineering for each enzyme–substrate pair. An alternative strategy involves enhancing the local concentration and interaction between the target protein and the modifying enzyme through an induced affinity. This can be achieved by introducing recruitment domains that direct the enzyme to its substrate.^38^ Specifically, incorporating a scaffolding domain into the target protein can recruit the modifying enzyme to the vicinity of the substrate, reducing off-target modifications and minimizing disruption to host cell viability. This strategy draws inspiration from the modular design of many natural enzymes, which often possess distinct domains: one for catalytic activity and another for substrate targeting. By mimicking this architecture, it is possible to improve reaction specificity and efficiency by bringing the enzyme and substrate into close proximity.^39^

In this study, we present an efficient, selective, and controlled method for the phosphorylation of recombinant silk proteins in *E. coli*. Our approach leverages a modified SpyCatcher/SpyTag protein–peptide pair as a recruitment system to induce proximity between the target protein and the kinase. This engineered spatial arrangement enhances substrate specificity and reduces the off-target activity. We demonstrate that this strategy improves host cell viability and enhances both the phosphorylation level and kinase selectivity. Importantly, the method is broadly applicable, here successfully implemented for two distinct types of spider silk proteins, dragline silk and aggregate silk, without requiring extensive optimization. Furthermore, we show that phosphorylation enhances the adhesive properties of the silk proteins, supporting its functional relevance in structural and bioadhesive materials.

## Materials and Methods

### Construction of Plasmids for Protein Expression

Synthetic genes coding for aggregate silk, Mouse kinase, and Mouse kinase with SpyTag­(A) were codon optimized for *E. coli* and ordered from GeneArt (Fisher Scientific), followed by subcloning into expression vectors, as detailed below. A list of constructs and full protein sequences is provided in Supporting Information (Figure S1, Table S1).

Two silk protein constructs were used as target proteins, each constructed into plasmid pRSFDuet1 with a kanamycin resistance gene and T7 promoter system inducible with isopropyl-β-D-thiogalactoside (IPTG). The cloning of *Araneus diadematus* dragline silk fibroin (ADF3) fused to SpyCatcher002, SilkTag, and HisTag, resulting in a plasmid named pSARSFDuet300, encoding the protein SpyCatcher002-ADF3-SilkTag-H6, has been reported before.^40^

A gene coding for a fragment from *Argiope trifasciata* aggregate silk spidroin 1 (AgSp1)^41^ (GenBank QDI78451.1) was inserted into pSARSFDuet300 plasmid using *Nhe*I and *Eco*RI sites, resulting in a plasmid named pSARSFDuet390, encoding the protein SpyCatcher002-AgSp1-SilkTag-H6.

A synthetic gene coding for a Mouse kinase was subcloned into plasmid pBAD with the ampicillin resistance gene and araBAD promoter inducible with l-arabinose (L-ara). Only the active domain from Nek1-like kinase from house mouse *Mus musculus* (Uniprot P51954) was used for the kinase construct (MK). For the induced proximity method, the aspartic acid involved in the formation of the isopeptide bond in SpyTag was mutated to alanine, and the modified peptide was fused to the N-terminus of MK (MK-ST).

### Small-Scale Optimization of Induction Conditions for Protein Expression

*E. coli* T7 Express cells (New England Biolabs) were transformed with the respective plasmids for protein expression. For coexpression, two plasmids with different antibiotic resistance genes were transformed into the cells. Transformed cells were plated on Lysogeny broth (LB) agar plates supplemented with 50 μg/mL kanamycin and/or 100 μg/mL ampicillin (final concentrations). The cells were inoculated into 7 mL of LB medium supplemented with 50 μg/mL kanamycin and/or 100 μg/mL ampicillin (final concentrations). The cultures were incubated at 37 °C with 220 rpm shaking until OD_600_ reached 0.3–0.5. Protein production was induced by adding 200 μM IPTG and/or 0.001%–0.04% l-arabinose (final concentrations) with varying time intervals between inductions, followed by incubation at 30 °C (ADF3) or 18 °C (AgSp1) with 220 rpm shaking for a total of 18 h, as specified in Figures 1D andS2. The cells were harvested by centrifugation at 15,000*g* for 5 min at room temperature (RT).

**Figure 1:**
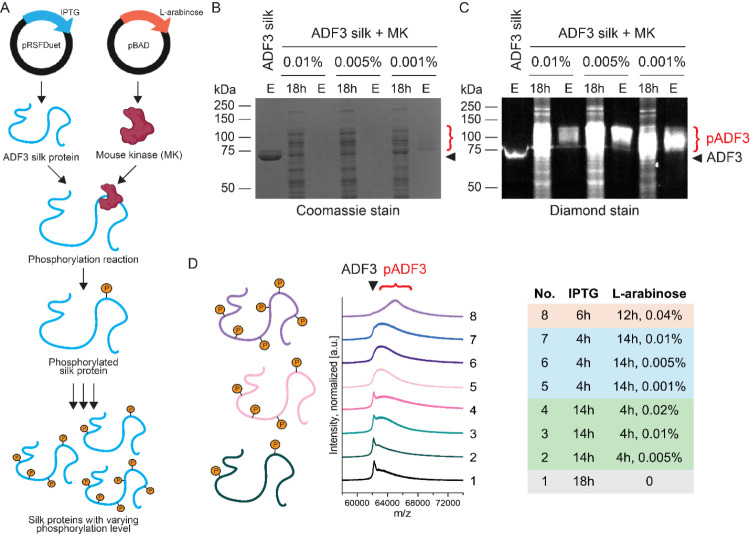
Efficient phosphorylation of dragline spider silk in *E. coli* by coexpression with a robust Mouse kinase. (A) Schematic overview of the two-plasmid coexpression system for phosphorylated ADF3 silk proteins. (B) Coomassie-stained and (C) Diamond-stained SDS-PAGE gels showing the coexpression of ADF3 silk protein and Mouse kinase. 18h stands for 18h postinduced whole cell lysate sample. E stands for elution. (D) MALDI-TOF analysis of controlled phosphorylation of ADF3 silk protein. Black arrows and red brackets indicate the expected apparent molecular weights of unmodified and phosphorylated ADF3, respectively.

The harvested cells were lysed with 200 μL of a B-PER solution (Fisher Scientific). The samples were purified by immobilized metal affinity chromatography (IMAC) using Nickel^2+^ ion coupled to nitrilotriacetic acid (Ni-NTA) spin columns (Qiagen) following the manufacturer’s protocol. Binding buffer (20 mM Imidazole, 300 mM NaCl, 20 mM Tris-HCl, pH 7.4), washing buffer (43 mM Imidazole, 300 mM NaCl, 20 mM Tris-HCl, pH 7.4), and elution buffer (250 mM Imidazole, 300 mM NaCl, 20 mM Tris-HCl, pH 7.4) were used for purification.

### Analysis of Cell Growth Rates

Precultures were prepared from the cells on LB plates by inoculating them into 7 mL of LB medium supplemented with 50 μg/mL kanamycin and/or 100 μg/mL ampicillin (final concentrations). The precultures were incubated at 30 °C with 220 rpm shaking overnight. Main cultures were prepared with 50 mL of LB media supplemented with 50 μg/mL kanamycin and/or 100 μg/mL ampicillin (final concentrations) and approximately 2 mL of preculture to achieve a starting OD_600_ of 0.05. The cultures were incubated at 37 °C with 220 rpm shaking until the OD_600_ was 0.2–0.3. The cultures were induced to 200 μM IPTG and/or 0.005% l-arabinose (final concentrations). The cultures were incubated at either 30 °C (ADF3) or 18 °C (AgSp1) with 220 rpm shaking for 7 h. OD_600_ was measured each hour.

### Large-Scale Protein Expression

Cells were inoculated into 45 mL of LB medium supplemented with 50 μg/mL kanamycin and/or 100 μg/mL ampicillin (final concentrations). The precultures were incubated at 37 °C with 220 rpm shaking for 6 h. Main cultures were prepared with 500 mL of EnPresso B 500 media (Enpresso GmbH) according to the manufacturer’s protocol, supplemented with 50 μg/mL kanamycin and/or 100 μg/mL ampicillin (final concentration), and induced to 200 μM IPTG and/or 0.005% l-arabinose (final concentrations). The cultures were incubated at either 30 °C (ADF3) or 18 °C (AgSp1) with 220 rpm shaking for 24 h. Alternatively, the production of AgSp1 and MK-ST was induced by adding 200 μM IPTG to induce the production of AgSp1 at time 0, followed by adding 0.005% l-arabinose for the induction of kinase production after 4 h incubation at 25 °C (final concentrations). Cells were incubated for 24 h at 25 °C prior to harvesting. All cells were harvested by centrifugation at 12,000×*g* for 15 min at RT.

Collected cells were resuspended in 3 mL of lysis buffer (50 mM Tris-HCl, 100 mM NaCl, 3 mM MgCl_2_, 1 mg/mL lysozyme, 0.01 mg/mL DNase I, 1 Pierce Protease Inhibitor Tablet (Fisher Scientific) per 50 mL buffer, pH 7.4) per 1 g of pellet and incubated at RT with 150 rpm for 1 h. The samples were sonicated (Qsonica 500) (1 min, 2 s pulse on/off, 40% amplitude) 3 times using a 1/8″ microtip (Qsonica) and centrifuged at 25,000×*g* for 30 min at RT. The supernatant was collected and heat-treated at 70 °C water bath for 10 min. The samples were centrifuged twice at 3,200×*g* for 15 min, discarding the pellet. Further purification was done by IMAC using Äkta purifier (Cytiva), two 5 mL HisTrap FF columns, binding buffer (20 mM imidazole, 300 mM NaCl, 20 mM Tris-HCl, pH 7.4), and elution buffer (250 mM imidazole, 300 mM NaCl, 20 mM Tris-HCl, pH 7.4). The elution fractions with the desired protein based on absorbance were collected. The buffer exchange was performed using Econo-Pac Desalting Gravity Flow Columns (Bio-Rad), following the manufacturer’s protocol, resulting in a sample volume of approximately 40 mL.

### Phosphoprotein Detection *via* Gel Electrophoresis

Whole cell samples were prepared by centrifuging 500 μL of cell culture 15,000×*g* for 2 min at RT. The acquired pellet was resuspended to 1× SDS loading dye, normalizing the samples by adding 10 μL of the dye per OD_600_ of 0.1. Elution samples were prepared by using 40 μL of the elution mixed with 10 μL of 4× SDS loading dye. Either 1.5 μL of Precision Plus Protein Dual Color Standard (Bio-Rad) or 5 μL of the sample was loaded on 10% SDS-PAGE gel.

The SDS-PAGE gels were stained with either Coomassie blue stain or Pro-Q Diamond Phosphoprotein Gel Stain (Invitrogen) (referred to as Diamond stain). The Coomassie-stained gel was imaged with a ChemiDoc XRS Imaging System (Bio-Rad). Diamond staining was done according to the manufacturer’s protocol, using lab-prepared destaining solution (20% acetonitrile, 50 mM sodium acetate, pH 4). The gel was imaged with the ChemiDoc MP Imaging System (Bio-Rad) with the Pro-Q Diamond program option.

### Western Blotting

SDS-PAGE gels were electroblotted to a nitrocellulose membrane (Merck) with Trans-Blot Turbo Transfer System (Bio-Rad). The membrane was blocked in 5% (w/v) fat-free milk in Tri*sec*-buffered saline with 0.1% Tween-20 (Sigma-Aldrich) (TBS-T) overnight at 4 °C with shaking. The membrane was incubated for 1 h in 1% (w/v) fat-free milk in TBS-T probed with either 6x-HisTag Monoclonal Antibody HRP (Invitrogen), Mouse Anti-Phosphoserine Antibody HRP (Sigma-Aldrich, Invitrogen), or Rabbit Anti-Phosphotyrosine-HRP Antibody (Invitrogen). After washing the membrane with TBS-T, Pierce ECL Western Blotting Substrate (Fisher Scientific) was used as a detection reagent. The membrane was imaged using the ChemiDoc MP Imaging System (Bio-Rad) with the Chemiluminescence program option.

### MALDI-TOF

The modification level in phosphorylated silk samples was analyzed using matrix-assisted laser desorption ionization time of flight (MALDI-TOF) (Bruker). Matrix for 2,5-DHAP dried droplet protocol (Bruker) was prepared by dissolving 7.6 mg of 2,5-dihydroxylactetophenone (2,5-DHAP) (Sigma-Aldrich) in 375 μL of ethanol and mixing with 125 μL of solution containing 18 mg/mL diammonium hydrogen citrate (Riedel-de Haen) in water. For analysis, 2 μL of elution sample from the small-scale expression test, 2 μL of prepared matrix, and 2 μL of 2% trifluoroacetic acid were mixed together until crystals were formed. On a ground steel plate, 1 μL of the mixture was added for analysis.

### Liquid Chromatography Mass Spectrometry

To study the protein phosphorylation sites, liquid chromatography mass spectrometry (LC-MS/MS) was performed for the purified protein samples of the control (ADF3 silk produced without kinase), ADF3 with low phosphorylation level (coexpressed with Mouse kinase), and ADF3 with high phosphorylation level (coexpressed with Mouse kinase fused with SpyTag­(A)). Each sample was prepared in 1 mg/mL (final concentration) in 50 mM Tris-HCl, pH 7. The data was processed by excluding the results with 5 or less phosphorylation counts per phosphorylation site in a peptide, to minimize false positives. Total phosphorylation count for a specific amino acid was obtained by adding together the counts from all of the peptides including the amino acid.

### Lap Shear Tests for Adhesion Studies

Protein samples in 50 mM NaCl buffer were concentrated to 50 mg/mL using Vivaspin 20 or Vivaspin 6 concentrators (Sartorius) with 10 kDa molecular weight cutoff (MWCO). For lap shear samples containing calcium, CaCl_2_ was added to a 10 mM final concentration. Lap shear tests for adhesion studies were performed by using glass slides (50 × 5 × 2 mm^3^) as substrates to measure the maximum failure force. The glass slides were soaked in soap solution (Hellmanex III, 2%) for 3 h, rinsed with ethanol and deionized water, and dried overnight in air at RT. From the concentrated samples, 4 μL of the protein solution was spread on the glass slide with a pipet. After 1 min of drying in air, another glass slide was placed on top of the spread sample with an overlapping area of 5 × 5 mm^2^, and the glass slides were fixed with clamps to ensure good contact. The samples were dried overnight at RT. Measurements were performed on an Instron 68TM-5 universal mechanical tester with a 5 kN loading cell and a loading rate of 1.5 mm/min, recording the load–displacement curve in the process. The adhesive strength was calculated by the maximum failure force divided by the sample overlap area.

### Circular Dichroism Spectroscopy

Secondary structure of the protein samples was studied using a JASCO J-1500 spectrometer. The purified proteins were diluted to a 0.18 mg/mL protein concentration. Samples were measured in either water, in 10 mM NaCl, or in 10 mM CaCl_2_. A quartz cuvette (Hellma) with a 1 mm path length was used for the sample. The samples were scanned 3 times with a baseline correction in each solution. A wavelength range from 250 to 180 nm was used together with a 1 nm bandwidth. Measurements were conducted at room temperature. Molar ellipticity was calculated from measured millidegrees according to eq 1, where m°, M, L, and C stand for millidegrees (mdeg), protein mean residual weight (g/mol), path length of cuvette (cm), and concentration (g/l).

1 $$m\mathrm{olar}\;e\mathrm{llipticity}=\frac{m^{{}^{\circ}}\cdot M}{10\cdot L\cdot C}$$

## Results and Discussion

### Production of Phosphorylated Silk Proteins by the Coexpression of Silk Protein and Kinase

As the first step toward controlled phosphorylation of silk protein, we wanted to establish efficient phosphorylation of silk proteins in *E. coli*. To achieve this, we took advantage of a coexpression platform for *E. coli* (Figure 1A).^42^ Two different plasmids were transformed into *E. coli*, one carrying a target silk protein and another kinase enzyme. The target protein was cloned into an IPTG-inducible pRSFDuet1 vector with kanamycin resistance, while the kinase was constructed in an l-arabinose-inducible pBAD vector with ampicillin resistance (Figure 1A). Our hypothesis was that the target protein would be phosphorylated by the kinase after translation, and the tight and controllable induction from the pBAD vector would facilitate the production of kinases that are often toxic for the host.^33,43^

We used as a target silk a fragment of the dragline silk protein ADF3 from the European garden spider (*Araneus diadematus*), called here ADF3.^44^ The ADF3 silk protein was fused to SpyCatcher002 ^45^ to improve solubility and enable the conjugation of silk fragments.^40,46^ As a kinase, we used the catalytic domain of a Mouse kinase that has been reported to have high efficiency and broad specificity.^47^

Coexpression of the kinase and silk protein resulted in phosphorylation of the silk, as visible from increased apparent and measured molecular weight shown on SDS-PAGE and by analysis with MALDI-TOF (Figure 1). Phosphorylated target proteins were poorly stained with Coomassie stain (Figure 1B). This is likely due to the negative charge caused by the addition of the phosphate groups, as the Coomassie stain binds to the hydrophobic and positively charged amino acids.^48^ Staining using phosphoprotein-specific Diamond stain confirmed phosphorylation and the associated molecular weight shift (Figure 1C). We expected to achieve heterogeneous phosphorylation of multiple sites, which was supported by the broad peak corresponding to phosphorylated silk proteins observed by analysis with MALDI-TOF (Figures 1D,S2). Phosphorylated silk proteins were thermostable, which allowed us to include a heat-precipitation step in the purification protocol (Figure S3).

We next wanted to investigate whether the phosphorylation level could be controlled by adjusting the induction level of the kinase through varying arabinose concentration and induction duration.

Indeed, increasing the arabinose concentration, which induces the production of kinase, leads to higher target protein phosphorylation levels (Figures 1D,S2). However, this increase is accompanied by a decline in cell viability, attributed to nonspecific modifications by the kinase. Adjusting the timing of the kinase induction affected the amount of unmodified silk that was left in the sample. A longer time interval of 14 h between the induction of silk and kinase led to an increased amount of unmodified silk, whereas inducing kinase only 4 h after the silk produced more modified target protein. We concluded that by using 0.005% l-arabinose for kinase induction for 14 h after an initial 4 h silk induction, most of the silk is modified with minimized effect on the cell viability.

### Induced Proximity Increases Phosphorylation Efficiency and Host Cell Viability

Although we could demonstrate efficient phosphorylation in *E. coli*, the broad specificity of the kinase resulted in the phosphorylation of host proteins in addition to the target protein, as visible from the diamond staining of the whole cell lysate (Figure 1C, “18h” lanes). In order to improve the specificity of kinase, we decided to employ an induced proximity approach that mimics the modular architecture frequently used by natural enzymes that utilizes one domain responsible for activity and another to achieve specificity *via* proximity.^39^ This strategy enables improving selectivity without the need for tedious optimization of the kinase to achieve specificity toward the target protein and/or specific sequence. To induce proximity of the substrate and kinase, we took advantage of protein/peptide pairs called Catcher/Tag pairs^49^ as recruitment domains. Catcher/Tag pairs were originally engineered for protein conjugation applications, since they can catalyze isopeptide bond formation between the two fragments (Figure 2A).^50^ However, by inactivating one of the reactive residues, Catcher/Tag pairs can be used as affinity tags (Figure 2B).^51^

**Figure 2:**
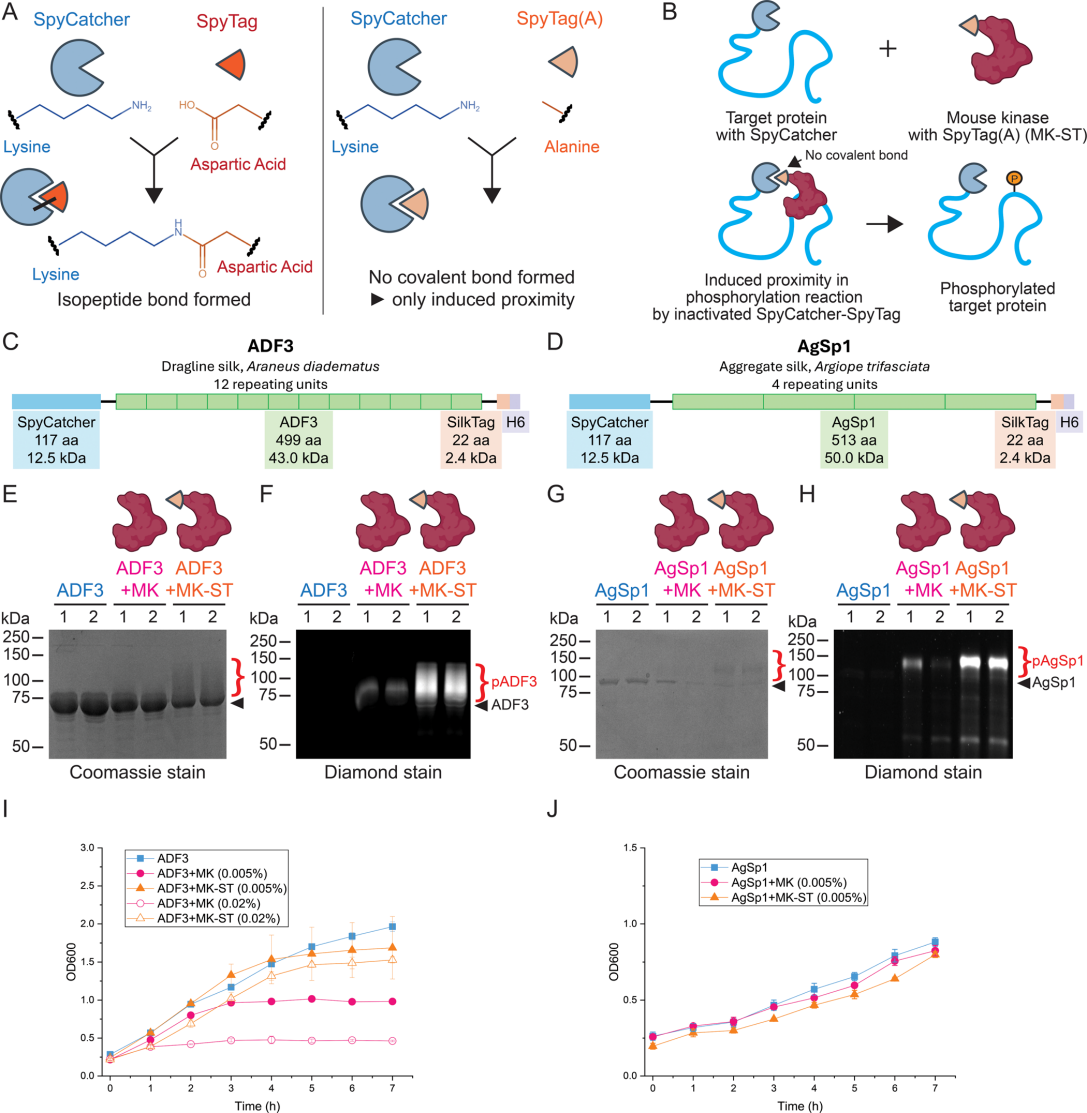
Induced proximity method for phosphorylation of dragline spider silk and aggregate spider silk in *E. coli* by coexpression with a robust Mouse kinase fused to SpyTag­(A). (A) Schematic overview of the inactivated SpyCatcher-SpyTag. (B) Induced proximity approach for silk protein phosphorylation. Schematic representation of (C) ADF3 dragline spider silk and (D) AgSp1 aggregate spider silk. (E) Coomassie-stained and (F) Diamond-stained SDS-PAGE gels for ADF3 silk protein, and (G) Coomassie-stained and (H) Diamond-stained SDS-PAGE gels for AgSp1 silk protein coexpressed with either Mouse kinase (MK) or Mouse kinase fused to SpyTag­(A) (MK-ST), 1 and 2 stand for duplicate expressions. Cell growth curves for (I) ADF3 silk protein and (J) AgSp1 silk protein. Black arrows and red brackets indicate the expected apparent molecular weights of the unmodified and phosphorylated target protein, respectively.

Here, we utilized the SpyCatcher/SpyTag peptide pair in which SpyTag was inactivated to prevent isopeptide bond formation by mutating catalytically active aspartic acid to alanine (Figure 2A). We fused the inactivated SpyTag­(A) peptide with the Mouse kinase (Table S1). As the target, we used the ADF3 silk protein fused with SpyCatcher002 (Figure 2C). In addition, we wanted to test the generality of the strategy and designed a construct coding for a 69.6 kDa fragment of aggregate silk spidroin 1 from *Argiope trifasciata* ^41^ as a fusion protein with SpyCatcher (Figure 2D) as an alternative target. Aggregate spider silks, which spiders use as a glue to capture prey, contain multiple phosphorylations, which, together with glycosylations, are considered to be responsible for the adhesive properties of the silk.^21^

Indeed, the induced proximity approach resulted in an increase in the phosphorylation level of ADF3, as observed from the higher apparent molecular weight of the target proteins on Coomassie-stained SDS-PAGE gels and in brighter protein bands on Diamond-stained SDS-PAGE gels (Figures 2E,F,S3, S4, and S5). The method could also be successfully applied to phosphorylate aggregate silk spidroin AgSp1 (Figures 2G,H,S4, S6). Furthermore, the phosphorylation of host proteins was reduced, as visible from fainter staining of the whole cell lysates on the Diamond-stained SDS-PAGE gels (Figure S4). While no (ADF3, Figure S4B) or very faint (AgSp1, Figure S4D) level of staining of the *E. coli* proteins was observed without the kinase, a high level of staining was observed upon coexpressing the kinase, and the level was significantly decreased after introducing an induced affinity approach (Figure S4). The differences between the background phosphorylations of ADF3 and AgSp1 are due to different exposure times. In addition, similarly to the coexpression with kinase without a fusion with SpyTag­(A), also in the case of the affinity-induced phosphorylation, the phosphorylation level could be tuned by adjusting induction levels and timing (Figure S5).

In the case of dragline silk ADF3, induced proximity clearly also reduced the toxic effects to the cell when compared to the use of only the active domain of the Mouse kinase, as higher cell densities were reached using the tagged kinase with 0.005% l-arabinose concentration (Figure 2I). Using the induced proximity approach, l-arabinose concentration could be increased to 0.02% without critically affecting the cell viability. On the contrary, cell viability was severely affected if the untagged kinase was produced.

Conversely, no significant difference in cell viability was observed when comparing untagged and tagged kinase samples expressing aggregate silk AgSp1 (Figure 2J). This lack of difference may be attributed to the low expression levels of the target protein AgSp1, resulting in fewer silk targets for the tagged kinase, thereby allowing the free enzyme to phosphorylate endogenous proteins within the cell. This highlights the need to optimize the l-arabinose concentration for kinase expression based on the target protein expression. Although using the SpyTag­(A)-fused kinase did not enhance the cell viability of AgSp1 samples, an increased phosphorylation level of the target proteins could be achieved (Figure 2G,H). Further optimization of aggregate spider silk expression could enhance cell viability results.

### Robust Mouse Kinase Phosphorylates Serines, Threonines, and Tyrosines

ADF3 dragline silk contains both serines and tyrosines, while aggregate silk AgSp1 contains serines, threonines, and tyrosines (Figure 3A, B). The Mouse kinase used is a dual activity kinase that can phosphorylate tyrosines as well as serines and threonines.^47^ In order to confirm the activity of the kinase toward both types of residues, we analyzed the modified samples by immunoblotting against phosphoserine and phosphotyrosine. Immunoblotting using anti-phosphoserine and anti-phosphotyrosine reveals that the Mouse kinase phosphorylates both serines and tyrosines, thus confirming its robustness toward the residues phosphorylated (Figures 3C–F,S6).

**Figure 3:**
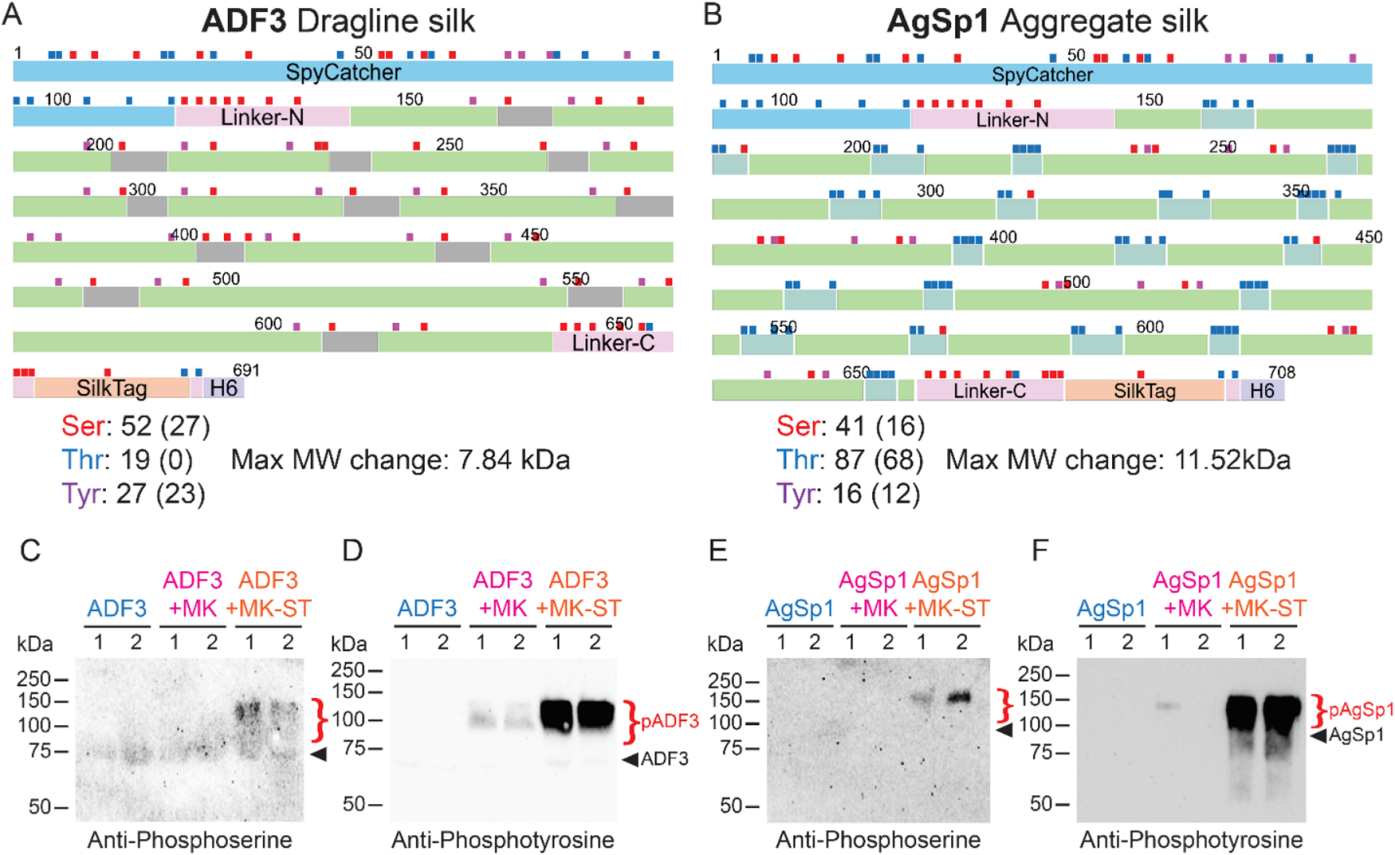
Phosphorylated residues in dragline spider silk and aggregate spider silk in *E. coli* by coexpression with a robust Mouse kinase fused to SpyTag­(A). Serine (red), threonine (blue), and tyrosine (purple) residues are highlighted in (A) ADF3 silk protein and (B) AgSp1 silk protein. Values in parentheses denote the number of residues in the silk domain of the construct. Maximal MW changes were calculated with 80 Da increase per phosphorylation. Immunoblotting of ADF3 silk protein (C, D) and AgSp1 silk protein (E, F) coexpressed with either Mouse kinase (MK) or Mouse kinase fused to SpyTag­(A) (MK-ST) with anti-phosphoserine and anti-phosphotyrosine, 1 and 2 stand for duplicate experiments. Black arrows and red brackets indicate the expected apparent molecular weights of the unmodified and phosphorylated target protein, respectively.

### Induced Proximity Approach Leads to a Significant Increase in the Number of Phosphorylation Sites

To obtain more detailed insights into the location and number of the modifications, we analyzed the phosphorylation of ADF3 dragline silk protein by LC-MS/MS (Figure 4). A control sample produced without a kinase showed phosphorylation at three serine residues and one tyrosine. This may be attributed either to low levels of intrinsic background phosphorylation by endogenous *E. coli* kinases or to artifacts arising from the analysis of silk protein fragments containing multiple repetitive sequences. A sample produced with the Mouse kinase active domain, *e.g*., without the induced proximity effect, shows a clear increase in the phosphorylation sites, now found in eight serines and two threonines. Finally, 35 phosphoserines, seven phosphothreonines, and ten phosphotyrosines were identified in the sample produced using the induced proximity approach, showing a significant increase in the phosphorylation level. The number of phosphoserines detected by proteomics was high compared to the results from immunoblotting with anti-phosphoserine (Figure 3C). This is likely due to the characteristics of the anti-phosphoserine antibody.

**Figure 4:**
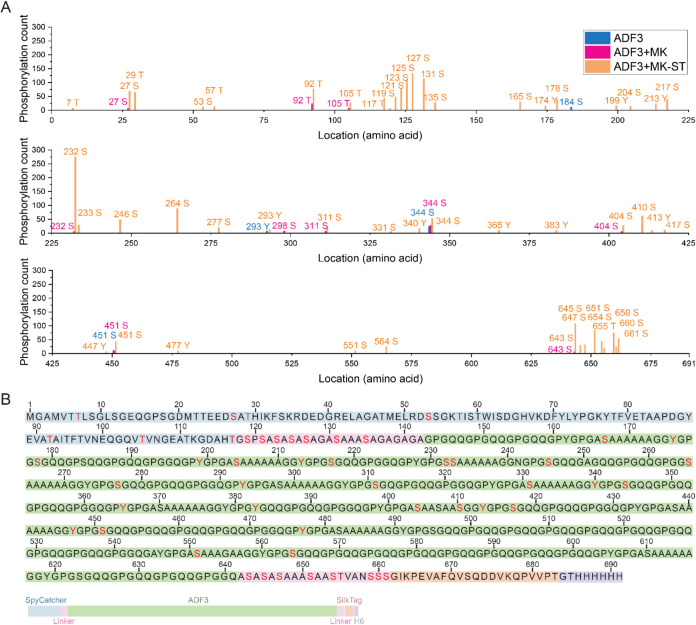
Phosphorylated residues in dragline spider silk analyzed with LC-MS/MS. (A) Phosphorylation of ADF3 silk protein. The peak height shows the phosphorylation count of each amino acid. (B) Amino-acid sequence for ADF3 silk protein construct. Phosphorylated residues in the ADF3+MK-ST sample are shown in red.

Twenty-eight out of the 50 (56%) possible phosphorylation sites in ADF3 silk were found to be modified in a subset of the peptides. Phosphorylated sites were not identical among different peptides, indicating heterogeneous populations of phosphorylated silk proteins in the sample. Based on MALDI-TOF data, the average number of phosphorylations in highly phosphorylated ADF3 silk is approximately 26 (Figure S7). Disordered serine-rich linker sequences were found to be phosphorylated at all possible sites. Eight out of 29 (27%) possible residues were phosphorylated in the globular SpyCatcher protein (Figure 4), in which phosphorylations were found on the loops and the surface of the protein (Figure S8).

### Phosphorylation Improves the Adhesion of Silk Proteins

Phosphorylation is considered to play a key role in the functionality of many protein-based adhesives.^11,21,24^ To test the adhesive properties of phosphorylated silks, we conducted lap shear adhesion tests on the phosphorylated and unmodified dragline and aggregate silks (Figures 5,S9,S10). Previous work on recombinant spider silk proteins has shown that they are effective adhesives on different substrates, including delignified cellulose and glass.^52,53^ Indeed, ADF3 dragline silk acted as an adhesive on glass, showing an adhesive strength of 2.96 ± 1.08 MPa. Instead, in the case of six out of the ten (60%) nonphosphorylated AgSp1 aggregate silk samples, the adhesive was broken below the measurement limit, and the adhesive strength could thus not be obtained in a reliable manner. We account for these difficulties due to the poor stability of unmodified aggregate silk, which may have resulted in a heterogeneous sample in the concentration phase.

**Figure 5:**
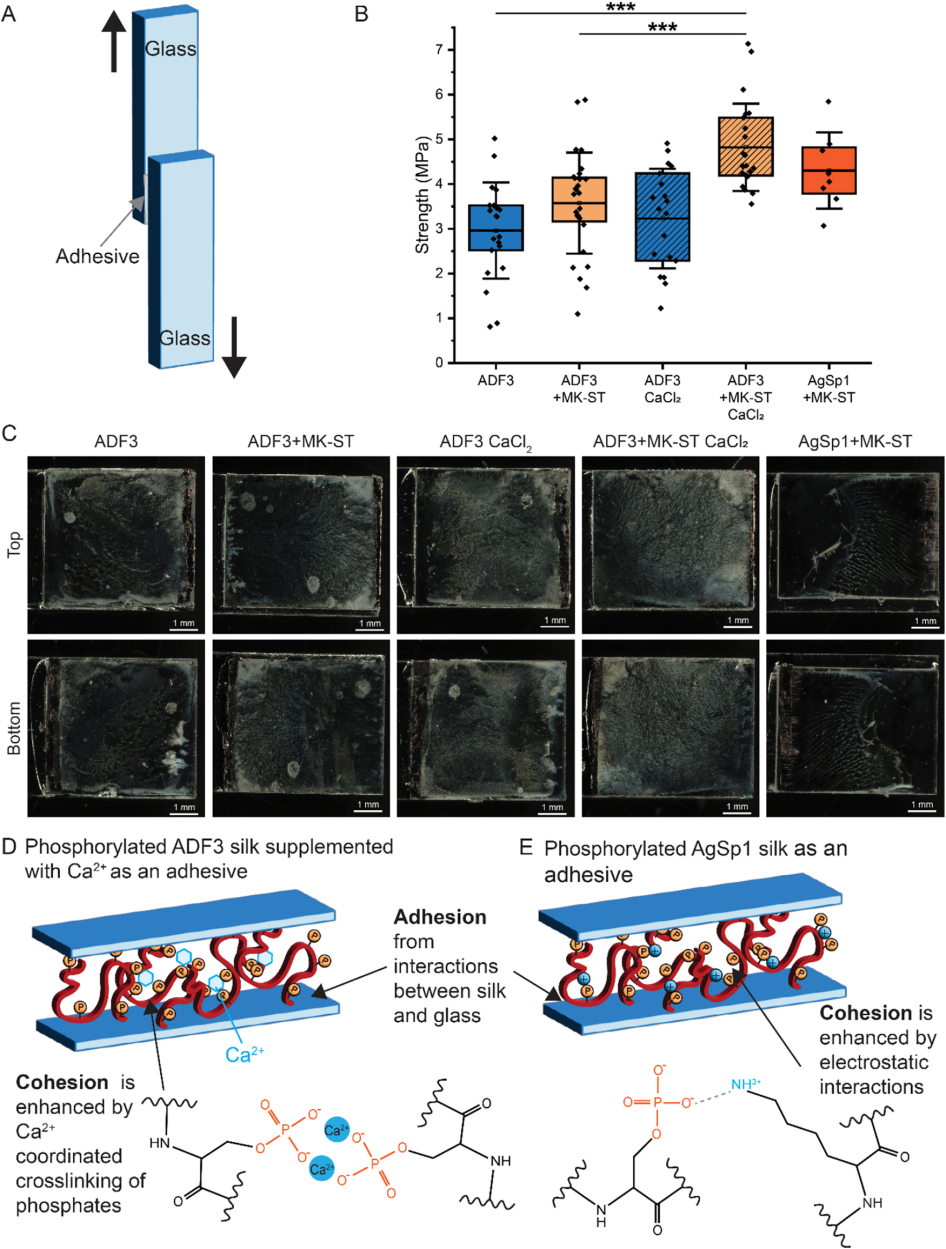
Phosphorylation enhances the adhesive properties of ADF3 spider silk. (A) Schematic figure of the lap shear test sample setup. (B) Analysis of the adhesive strength of unmodified and phosphorylated ADF3 dragline spider silk, with no salt, and with 10 mM CaCl_2_, as well as of the adhesive strength of phosphorylated AgSp1 spider aggregate silk. The distribution of the individual measurements is shown. The mean is shown as a black line. Whiskers show standard deviation. Three stars indicate *p* < 0.001. Samples broken below the measurement limit or while loading the equipment are excluded from the data. (C) Representative light microscope images of the fracture surfaces (more images are shown in Figure S7). (D) Schematic presentation of a possible adhesion mechanism of phosphorylated silk supplemented with Ca^2+^ ions. (E) Schematic presentation of a possible adhesion mechanism of the phosphorylated aggregate silk.

Phosphorylation was observed to slightly improve the adhesion of ADF3 silk since the adhesive strength of phosphorylated dragline silk was 3.57 ± 1.13 MPa. In line with these results, the adhesive strength of ADF3 with a low level of phosphorylation was between those of the unmodified and heavily phosphorylated ADF3 silks (Figure S10). In addition, we were able to obtain data for the phosphorylated aggregate silk, which showed an adhesive strength of 4.30 ± 0.85 MPa. MALDI-TOF analysis revealed a higher average modification level in phosphorylated aggregate silk samples compared to phosphorylated dragline silk samples, potentially explaining the greater adhesion strength observed in the aggregate silk (Figure S11). The addition of calcium ions slightly enhanced the adhesiveness of unmodified dragline spider silk, which showed an adhesive strength of 3.23 ± 1.11 MPa, whereas the observed adhesive strength for phosphorylated dragline silk with calcium ions was further increased to 4.82 ± 0.98 MPa. It is notable that 14/35 (40%) of the samples containing unmodified dragline silk failed below the measurement limit, while out of the phosphorylated sample, 7/35 (20%), and of the phosphorylated silk samples with CaCl_2_, only 3/25 (12%) could not be measured. For aggregate silk, 6/10 (60%) of samples containing unmodified silk failed before measurement, while all 8/8 (100%) samples for phosphorylated silk could be measured.

The analysis of the fracture surfaces of the samples broken from the adhesive area shows adhesive on both surfaces (Figures 5C,S12). This indicates that the mode of failure was cohesive or mixed. In six out of the 25 samples of phosphorylated silk supplemented with Ca^2+^ ions and in four out of the eight phosphorylated aggregate silk samples, the failure mode was substrate failure, since the glass substrate broke before the adhesive failed (Figure S12). Our interpretation is that both the modified and unmodified silks have strong adhesion on glass, while the limited mechanical stability of the adhesive led to adhesive failure. Similar results were obtained in a previous work on unmodified and DOPA-modified dragline silk adhesives.^52^

Multiple covalent and noncovalent strategies have been applied to address the cohesive failure of bioadhesives.^54,55^ Among these, Ca^2+^ ion-mediated cross-linking of phosphorylated polymers has been applied to improve the cohesion of phosphoserines and phosphoserine-mimicking synthetic polymers,^56^ as well as in the case of synthetic phosphoserines supplemented with Ca^2+^ ions.^56^ The observed improvement in the adhesive strength of phosphorylated silks in comparison to the unmodified silk could arise from an increase in electrostatic interactions between the silk molecules, while metal-coordinated interactions mediated by the Ca^2+^ ions are hypothesized to have further increased cohesion by cross-linking the silk molecules.

The analysis of protein secondary structures by circular dichroism spectroscopy shows that neither phosphorylation nor the addition of calcium causes major conformational changes in ADF3 silk, which remains mainly disordered (Figure S13). The addition of 10 mM CaCl_2_ or NaCl did not cause conformational changes in the unmodified silk. A small shift in the peak around 199 nm was observed in the phosphorylated silk sample supplemented with 10 mM CaCl_2_. We account for this change to the binding of calcium ions on the silk, causing minor local changes (Figure 5D). Unfortunately, we were not able to reliably analyze Young’s modulus due to the slipping of the samples—which commonly occurs in lap shear tests—causing experimental discrepancies in the observed elongation values (Figure S9 and Supporting data file). Unlike ADF3 dragline silk, aggregate silk AgSp1 is rich in lysine (Table S1). A possible explanation is that electrostatic interactions between negatively charged phosphate groups and positively charged side chains increased the cohesion of the silk polymers without the addition of Ca^2+^ ions (Figure 5E).

Covalent cross-linking, achievable through methods such as click chemistry, photo-cross-linking, or diortho-phthalaldehyde (Dopa)-based reactions, typically results in high bond energies.^57^ In contrast, while noncovalent cross-linking is generally less stable, it can reform dynamically,^57^ enabling properties such as self-healing and injectability.^54^ Dopa-based chemistries can also facilitate noncovalent interactions and have been widely used in the development of adhesives with remarkable mechanical performance.^58,59^ Other noncovalent strategies include coacervation, enhanced hydrogen bonding, and polymer chain entanglement.^52,54,60^ Among these, Ca^2+^ ion-mediated cross-linking of phosphorylated recombinant proteins offers a versatile approach to noncovalent cross-linking. This method provides advantages such as reversibility, tunability through controlled phosphorylation, and broad applicability to proteins containing serine, threonine, or tyrosine residues.

Given the robustness of the kinase used and the modularity of the Catcher/Tag-based induced affinity system, the method presented here holds the potential for broad applicability across a variety of recombinant proteins. We propose that this method holds particular promise for applications involving underwater adhesives and biomineralization proteins.

Moreover, affinity domains such as Catcher/Tag pairs can be employed to enhance the specificity of other single-step enzymatic post-translational modifications, including proline or lysine hydroxylation and acetylation. In this study, the Catcher domain was fused to the target proteins. While this fusion may limit applications requiring native target proteins, this limitation could be addressed by alternative strategies. For example, the orientation of the fusion can be reversed, attaching the Catcher domain to the kinase and the smaller Tag peptide to the target protein, or a proteolytic cleavage site can be introduced between the Catcher and the target protein to enable postreaction removal of the fusion domain. Additionally, using kinases with improved substrate specificity compared with the Mouse kinase employed here could further enhance selectivity for particular residues or sequence motifs.

## Conclusions

We report here a scaffolding coexpression method for the efficient, selective, and controlled polyphosphorylation of recombinant silk proteins in *E. coli*. This approach is anticipated to be broadly applicable to a variety of target proteins without requiring extensive kinase optimization, thereby addressing a major bottleneck in the production of recombinant phosphoproteins. Beyond phosphorylation, the induced proximity coexpression method can be extended to facilitate the production of other post-translationally modified proteins in *E. coli*, which inherently lacks the complex post-translational modification machinery of eukaryotic systems. The adaptability to a wide range of substrates and modification types of our coexpression scaffolding methods represents a powerful tool that can engineer biomaterials inspired by nature, particularly for proteins involved in biomineralization, underwater adhesion, and bone cements.

Our findings show that phosphorylation improved the adhesive properties of the silk proteins and offered potential for cross-linking protein chains by Ca^2+^ to enhance the adhesiveness, reinforcing the role of the post-translational modification in tuning the functional performance of silk proteins. We further anticipate that other modifications, especially glycosylations commonly found in aggregate silks,^41^ could provide additional improvements in adhesiveness.

These results underscore the importance of incorporating post-translational modifications when studying and replicating naturally engineered structural proteins. Despite the technical challenges associated with their detection and synthetic replication, such modifications are crucial for achieving the functional complexity and performance seen in biological materials.

## Supplementary Material







## Notes

Supporting Figures and Table (PDF). The Supporting Information is available free of charge at https://pubs.acs.org/doi/10.1021/acs.biomac.5c01431.

Schematic illustrations of the constructs used; amino-acid sequences of the constructs used; heterogenous phosphorylation of ADF3 silk; purification of phosphorylated silk proteins by heat precipitation; decreased background phosphorylation with the induced proximity approach; effect of induction level on the phosphorylation using induced-proximity approach; analysis of phosphorylated AgSp1 by diamond staining and immunoblotting; MALDI-TOF analysis for unmodified and modified ADF3; phosphorylation sites in SpyCatcher; representative load-displacement curves; adhesion strengths of ADF3 silk with different phosphorylation levels; MALDI-TOF analysis for unmodified and modified AgSp1; representative light microscope images of the fractured surfaces; and secondary structure analysis of unmodified and modified ADF3 silk. (PDF)
Raw data from MS/MS (XLSX)
Raw data from lap shear tests (XLSX)

## Abbreviations

2,5-DHAP: 2,5-Dihydroxylactetophenone
ADF3: *Araneus diadematus* dragline silk fibroin
AgSp1: *Argiope trifasciata* aggregate silk spidroin 1
H6: HisTag
IMAC: Immobilized metal affinity chromatography
IPTG: isopropyl-β-D-thiogalactoside
L-ara: l-arabinose
LB: Lysogeny broth
MALDI-TOF: Matrix-assisted laser desorption ionization time of flight
MK: Mouse kinase
MK-ST: Mouse kinase with SpyTag­(A)
LC-MS/MS: liquid chromatography mass spectrometry
Ni-NTA: Nickel^2+^ ion coupled to nitrilotriacetic acid
PTM: post-translational modification
RT: room temperature
TBS-T: Tri*sec*-buffered saline with 0.1% Tween-20
