# Supplementary material for: Induced Proximity Approach Enables the Recombinant Production of Polyphosphorylated Silk Proteins with Improved Adhesiveness
Source: Biomacromolecules. 2025 Nov 25;26(12):8594–605. doi: 10.1021/acs.biomac.5c01431 (PMC12690576; doi:10.1021/acs.biomac.5c01431)
Supplement: Supplementary file 1 [file bm5c01431_si_001.pdf]

Supplementary document

Supporting Information: Induced proximity approach enables the recombinant production of polyphosphorylated silk proteins with improved adhesiveness

*Nea B. Möttönen[1], Ruxia Fan[1], Stefania Aspholm-Tsironi[1], Salla Keskitalo[2], Antti Tuhkala[2], Markku Varjosalo[2], A. Sesilja Aranko[1]**

[1] Department of Bioproducts and Biosystems, School of Chemical Engineering, Aalto University, 02150 Espoo, Finland

[2] Institute of Biotechnology and Helsinki Institute of Life Science, University of Helsinki, 00014 Helsinki, Finland

*E-mail: sesilja.aranko@aalto.fi

Table S1. Amino-acid sequences of the constructs used.

Protein construct	Amino acid sequence
**ADF3**<br><br>**SpyCatcher2-ADF3-SilkTag-H6**	MGAMVTTLSGLSGEQGPSGDMTTEEDSATHIKFSKRDEDGRELAGATMELRDSSGKTISTWISDGHVKDFYLYP<br>GKYTFVETAAPDGYEVATAITFTVNEQGQVTVNGEATKGDAHTGSPSASASASAGASAAASAGAGAGAGPGQ<br>QGPGQQGPGQQGPYGPGASAAAAAAGGYGPGSGQQGPSQQGPGQQGPGGQGPYGPGASAAAAAAGGYGP<br>GSGQQGPGGQGPYGPGSSAAAAAAGGNGPGSGQQGAGQQGPGQQGPGGSAAAAAAGGYGPGSGQQGPGQ<br>QGPGGQGPYGPGASAAAAAAGGYGPGSGQGPGQQGPGGQGPYGPGASAAAAAAGGYGPGSGQQGPGQQG<br>PGQQGPGGQGPYGPGASAAAAAAGGYGPGYGQQGPGQQGPGGQGPYGPGASAASAASGGYGPGSGQQGPG<br>QQGPGGQGPYGPGASAAAAAAGGYGPGSGQQGPGQQGPGQQGPGQQGPGGQGPYGPGASAAAAAAGGYG<br>PGSGQQGPGQQGPGQQGPGQQGPGQQGPGQQGPGQQGPGQQGPGQQGPGGQGAYGPGASAAAGAAGGY<br>GPGSGQQGPGQQGPGQQGPGQQGPGQQGPGQQGPGQQGPGQQGPYGPGASAAAAAAGGYGPGSGQQGPG<br>QQGPGQQGPGGQASASASAAASAASTVANSSSGIKPEVAFQVSQDDVKQPVVPTGTHHHHHH
**AgSp1**<br><br>**SpyCatcher2-AgSp1-SilkTag-H6**	MGAMVTTLSGLSGEQGPSGDMTTEEDSATHIKFSKRDEDGRELAGATMELRDSSGKTISTWISDGHVKDFYLYP<br>GKYTFVETAAPDGYEVATAITFTVNEQGQVTVNGEATKGDAHTGSPSASASASAGASAAASAGAGAGAGPDG<br>KPLPIEPAGPGTTPGTVTGPDGKPKKFVLPKGAFTTPGSIPGPDGKPIHVQPAGPGTTPGAQTGPDGKINKLVVP<br>TTTTPKGPVGPGGMPLSPYSPQGPGGQPMYPFGPGSPYGPGEQTTTTPIPGPDGKPLPIEPAGPGTTPGTVTGPD<br>GKPKKFVLPKGAFTTPGSIPGPDGKPIHVEPAGPGTTPGAQTGPDGKINKLVVPTTTTPKGPVGPGGMPLSPYSP<br>QGPGGQPMYPFGPGSPYGPGEQTTTTPIPGPDGKPLPIEPAGPGTTPGTVTGPDGKPKKFVLPKGAFTTPGSIPG<br>PDGKPIHVEPAGPGTTPGAQTGPDGKINKLVVPTTTTPKGPVGPGGMPLSPYSPQGPGGQPMYPFGPGSPYGP<br>GEQTTTTPIPGPDGKPLPIEPAGPGTTPGTVTGPDGKPKKFVLPKGAFTTPGSIPGPDGKPIHVEPAGPGTTPGA<br>QTGPDGKINKLVVPTTTTPKGPVGPGGMPLSPYSPQGPGGQPMYPFGPGSPYGPGEQTTTTPIPASASASAAAS<br>AASTVANSSSGIKPEVAFQVSQDDVKQPVVPTGTHHHHHH
**Mouse kinase**<br><br>**MK**	MGSEKYVRLQKIGEGSFGKAVLVKSTEDGRHYVIKEINISRMSDKERQESRREVAVLANMKHPNIVQYKESFEEN<br>GSLYIVMDYCEGGDLFKRINAQKGTLFQEDQILDWFVQICLALKHVHDRKILHRDIKSQNIFLTKDGTVQLGDF<br>GIARVLNSTVELARTCIGTPYYLSPEICENKPYNNKSDIWALGCVLYELCTLKHAFEAGNMKNLVLKIISGSFPPVS<br>PHYSYDLRSLLSQLFKRNPRDRPSVNSILEKGFIAKRIELE
**Mouse kinase with SpyTag(A)**<br><br>**MK-ST(A)**	MAHIVMV**A**AYKPTKSARASASAGASAAASAGAGAGAASEKYVRLQKIGEGSFGKAVLVKSTEDGRHYVIKEINI<br>SRMSDKERQESRREVAVLANMKHPNIVQYKESFEENGSLYIVMDYCEGGDLFKRINAQKGTLFQEDQILDWFV<br>QICLALKHVHDRKILHRDIKSQNIFLTKDGTVQLGDFGIARVLNSTVELARTCIGTPYYLSPEICENKPYNNKSDI<br>WALGCVLYELCTLKHAFEAGNMKNLVLKIISGSFPPVSPHYSYDLRSLLSQLFKRNPRDRPSVNSILEKGFIAKRIE

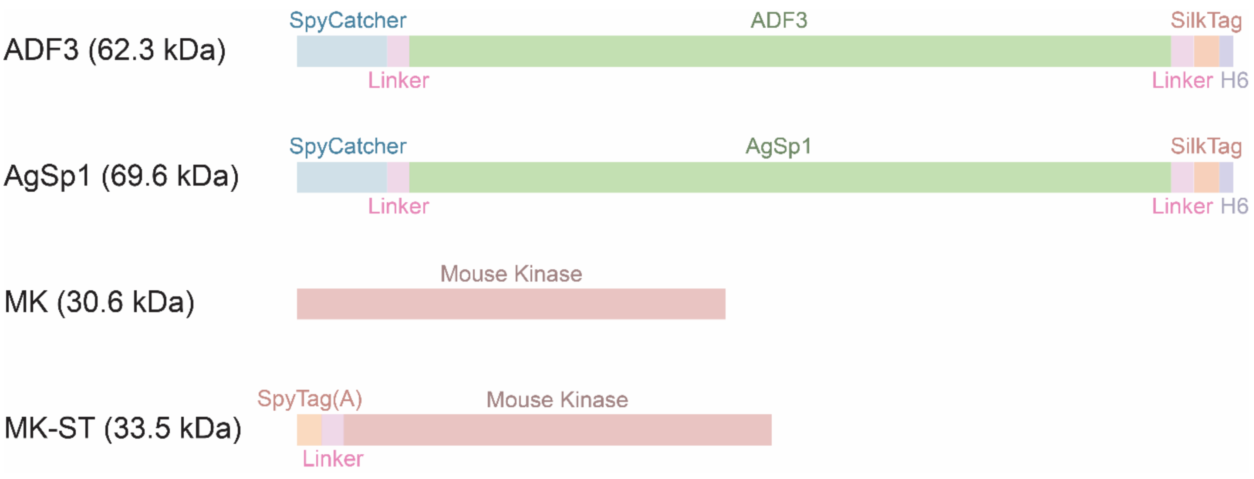

Figure S1. Schematic illustrations of the constructs used.

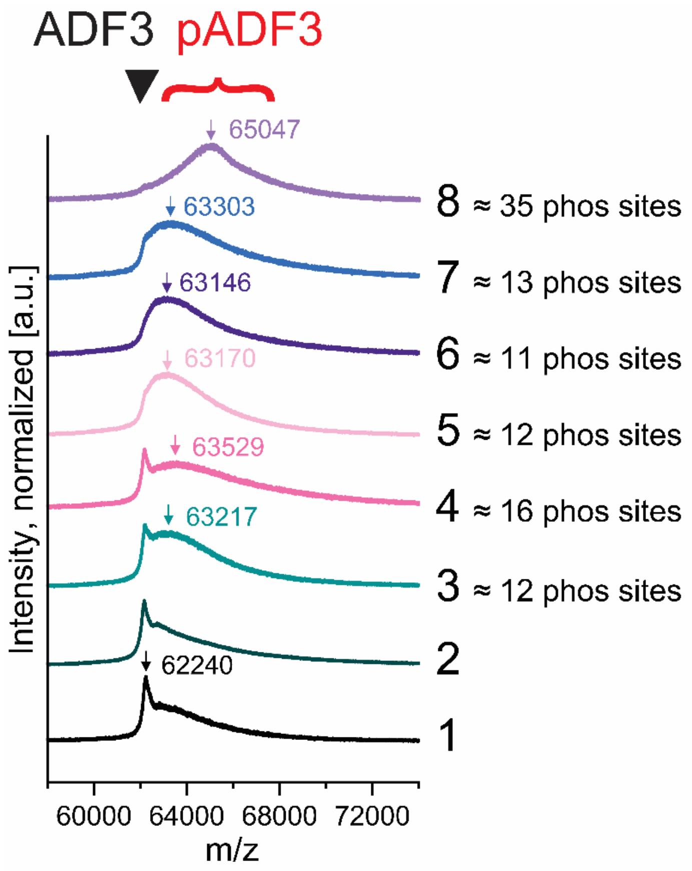

No.	IPTG	L-arabinose
8	6h	12h, 0.04%
7	4h	14h, 0.01%
6	4h	14h, 0.005%
5	4h	14h, 0.001%
4	14h	4h, 0.02%
3	14h	4h, 0.01%
2	14h	4h, 0.005%
1	18h	0

Figure S2. Heterogenous phosphorylation of ADF3 silk phosphorylated by MK in LB media at 30 °C, while inducing kinase either 4, 6 or 14 hours after the induction of silk. MALDI-TOF analysis of the phosphorylation level of ADF3 with theoretical MW of 62178.08 Da without M1. Highest point of the phosphorylation peak has been evaluated by eye. The phosphorylation sites of size 80 Da have been calculated from the difference between phosphorylation peak and the unmodified ADF3 peak in sample 1. Black arrow and red bracket indicate the expected apparent molecular weights of unmodified and phosphorylated ADF3, respectively.

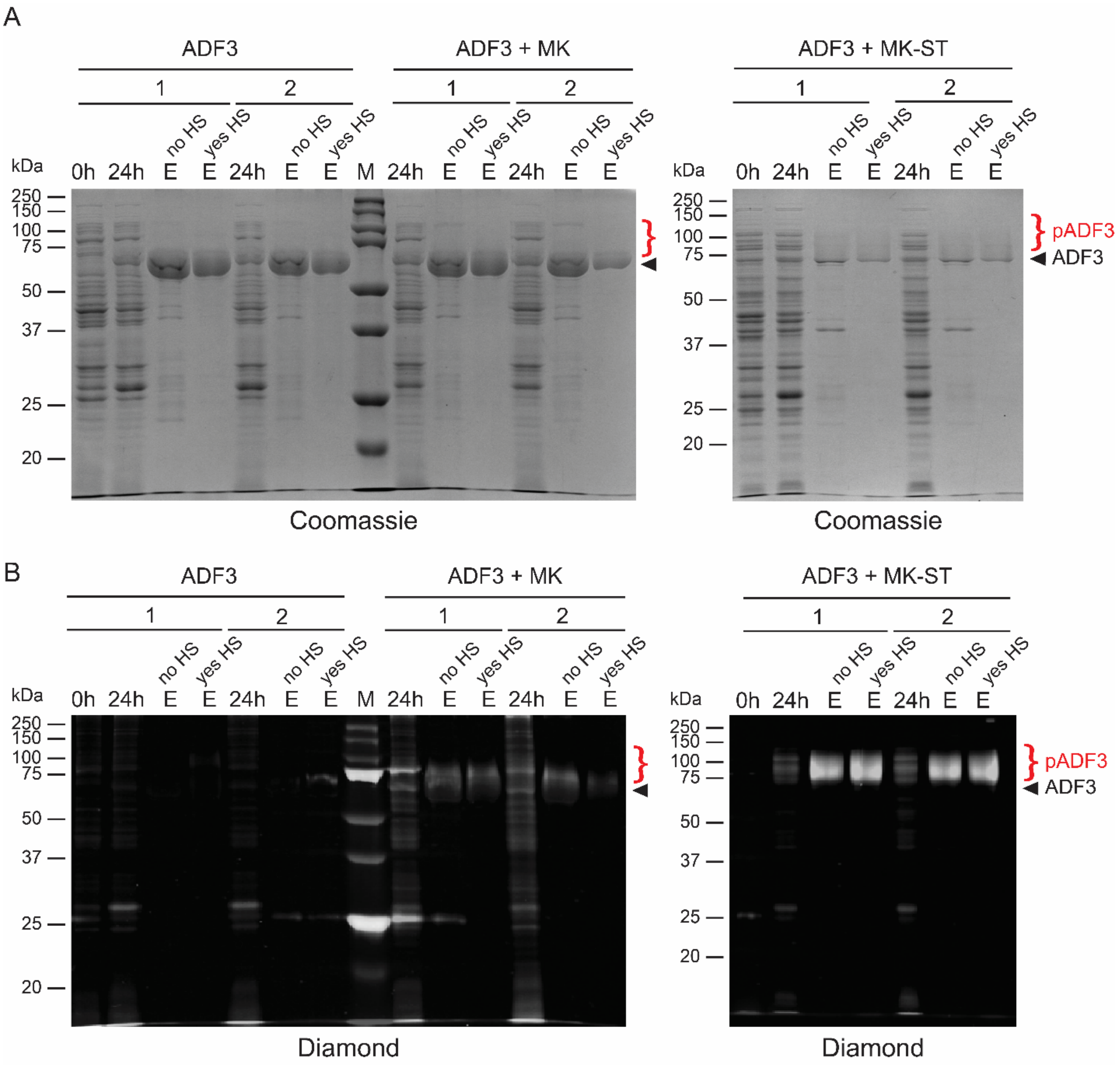

Figure S3. SDS-PAGE gels with Coomassie (A) and Diamond staining (B) for ADF3 and MK/MK-ST co-expression in Enpresso media, using 200 µM IPTG and 0.005% L-arabinose for induction (done at the same time) and incubating at 30 ˚C for 24 h. M stands for protein Marker. 0h and 24h stand for samples taken prior induction and after 24 h incubation, respectively. E stands for elution. 'No HS' and 'yes HS' stand for elution with and without heat

shock purification under 70 ˚C water bath for 10 min. The bands corresponding to unmodified and phosphorylated ADF3 silks are indicated with black arrows and red brackets, respectively.

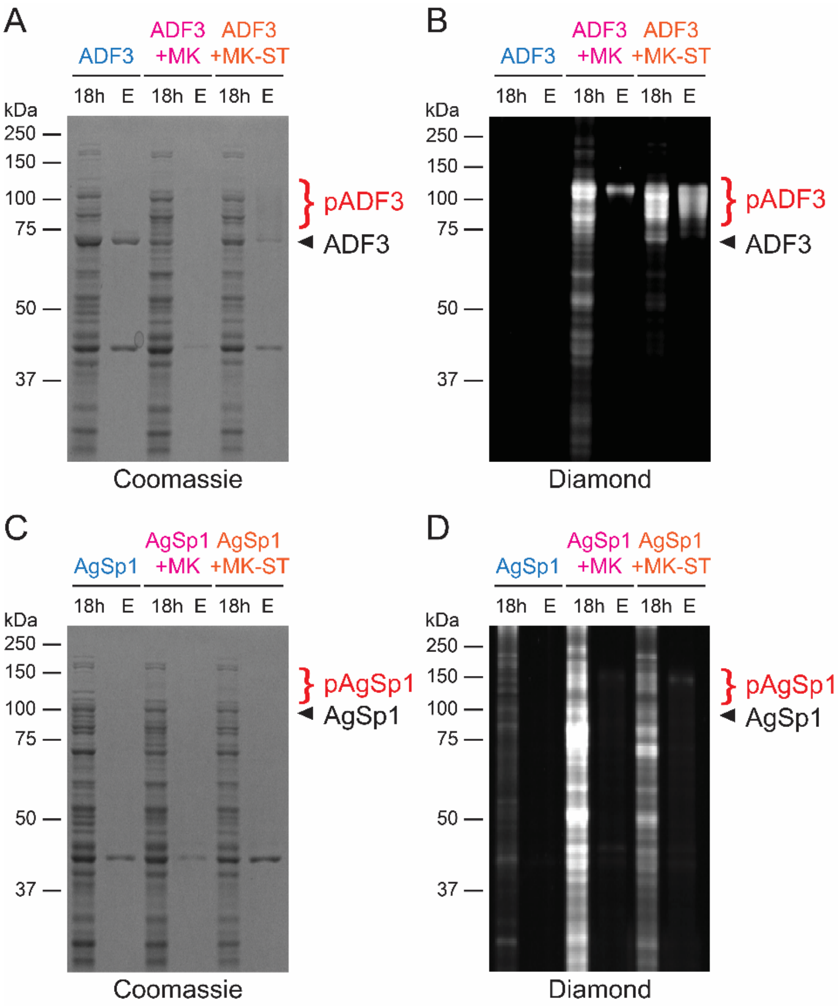

Figure S4. Decreased background phosphorylation with induced proximity approach. SDS-PAGE gel analysis of ADF3 and MK/MK-ST co-expression stained Coomassie (A) and Diamond staining (B). Co-expression was done in LB media, using 200 μM IPTG and 0.005% L-arabinose for induction (while inducing kinase 4 hours after the induction of silk) and incubating at 30 ˚C for 18 h. SDS-PAGE gels with Coomassie (C) and Diamond staining (D) for AgSp1 and

MK/MK-ST co-expression in LB media, using 200 µM IPTG and 0.005% L-arabinose for induction (while inducing kinase 4 hours after the induction of silk) and incubating at 18 ˚C for 18 h. The bands corresponding to unmodified and phosphorylated silks are indicated with black arrows and red brackets, respectively.

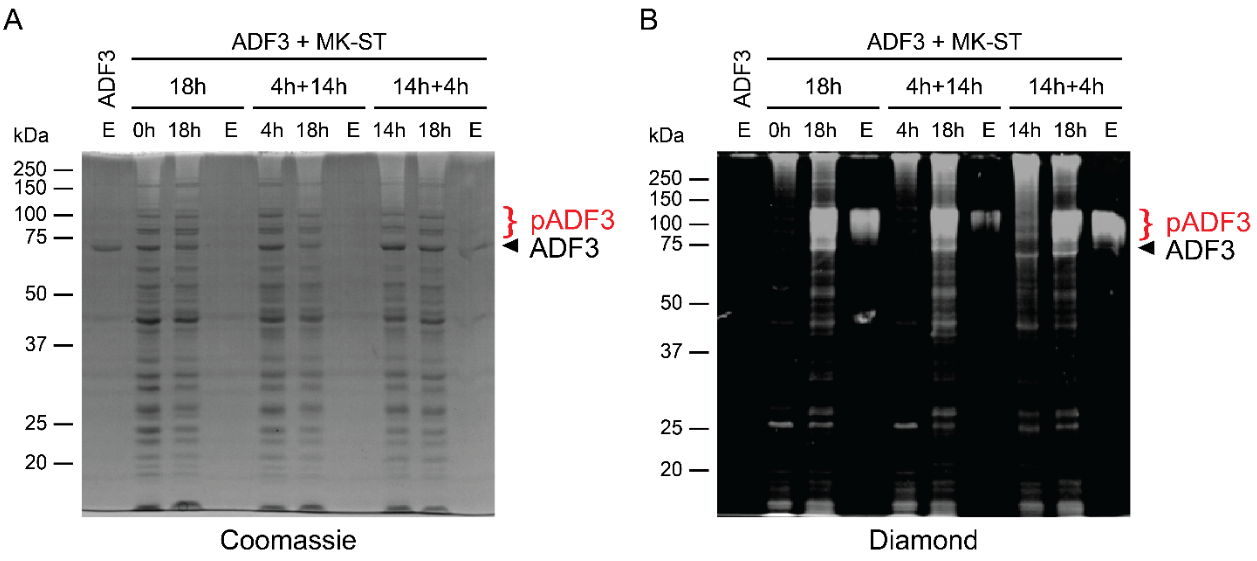

Figure S5. Co-expression of ADF3 silk and Mouse kinase with SpyTag (MK-ST). (A) SDS-PAGE gels with Coomassie (A) and Diamond staining (B) for ADF3 silk and MK-ST co-expression in LB media, using 200 µM IPTG and 0.005% L-arabinose and incubating at 30 ˚C for 18 h. Kinase has been induced either at the same time as silk (18 h), 4 hours after silk or 14 hours after silk. The bands corresponding to unmodified and phosphorylated silks are indicated with black arrows and red brackets, respectively.

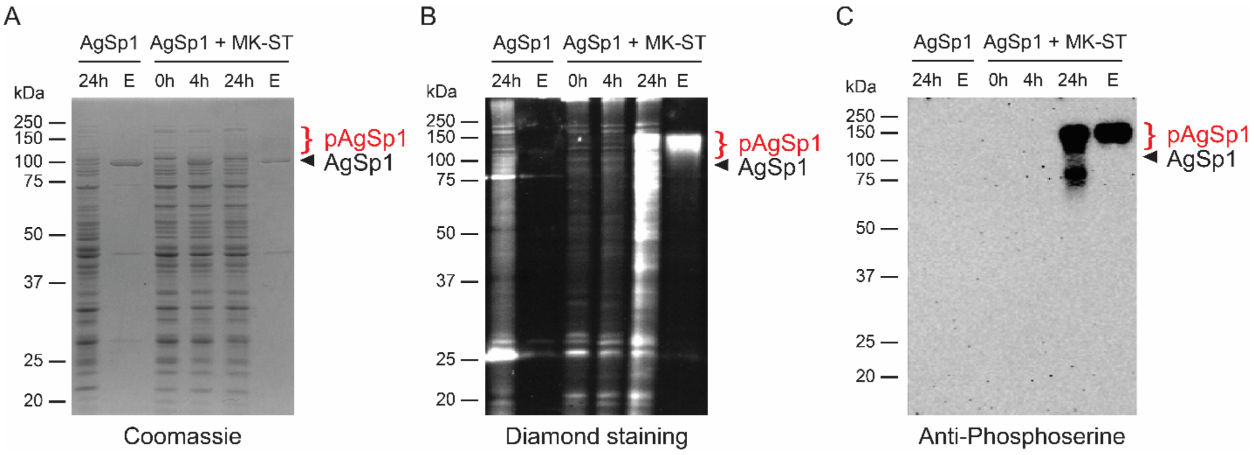

Figure S6. Analysis of the production of AgSp1 aggregate silk phosphorylated by MK-ST in Enpresso media at 25 ˚C, while inducing kinase 4 hours after the induction of silk. Analysis of production and phosphorylation levels of AgSp1 phosphorylated by MK-ST on SDS-PAGE gels stained with Coomassie (A) or Diamond staining (B), as well as analysed by immunoblotting using Anti-Phosphoserine antibody (C). E stands for elution. The bands corresponding to unmodified and phosphorylated AgSp1 aggregate silks are indicated with black arrows and red brackets, respectively.

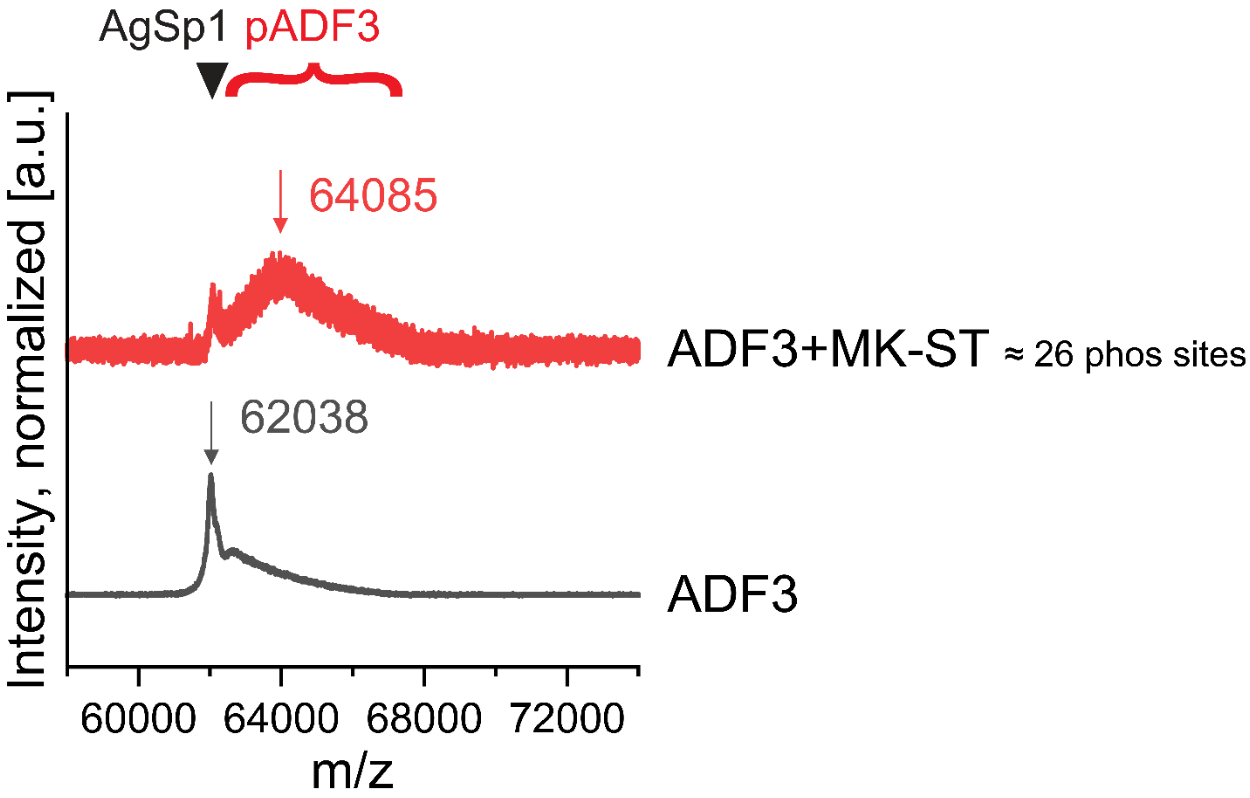

Figure S7. Co-expression of ADF3 silk and Mouse kinase with SpyTag (MK-ST). A) MALDI-TOF analysis for unmodified ADF3 with theoretical MW of 62178.08 Da without M1. B) MALDI-TOF analysis for co-expression of ADF3 with MK-ST using 18h induction sample. Black arrow and red bracket indicate the expected apparent molecular weights of unmodified and phosphorylated ADF3, respectively.

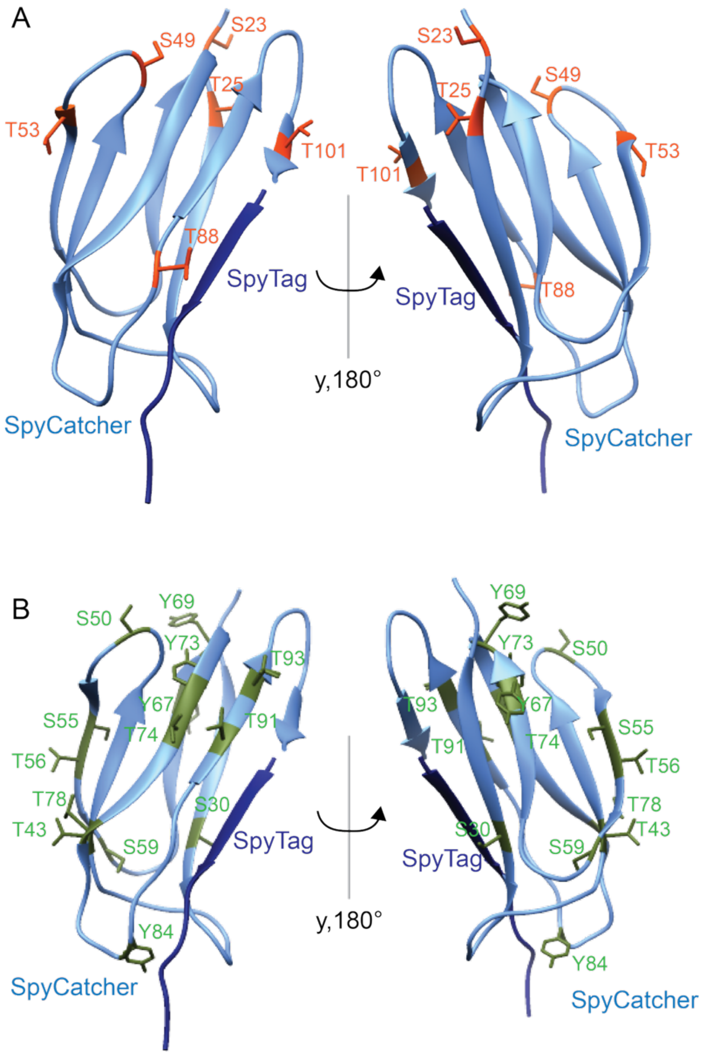

Figure S8. Phosphorylation of SpyCatcher. (A) Phosphorylated sites identified by MS-MS (in red) are located on the surface of the protein. (B) Potential phosphorylation sites (Ser/Thr/Tyr residues) on which phoshorylations were not observed (in green). Illustrated SpyCatcher structure (PDB: 4MLI) corresponds to amino acid residues from S26 to V106 in ADF3 silk protein construct.

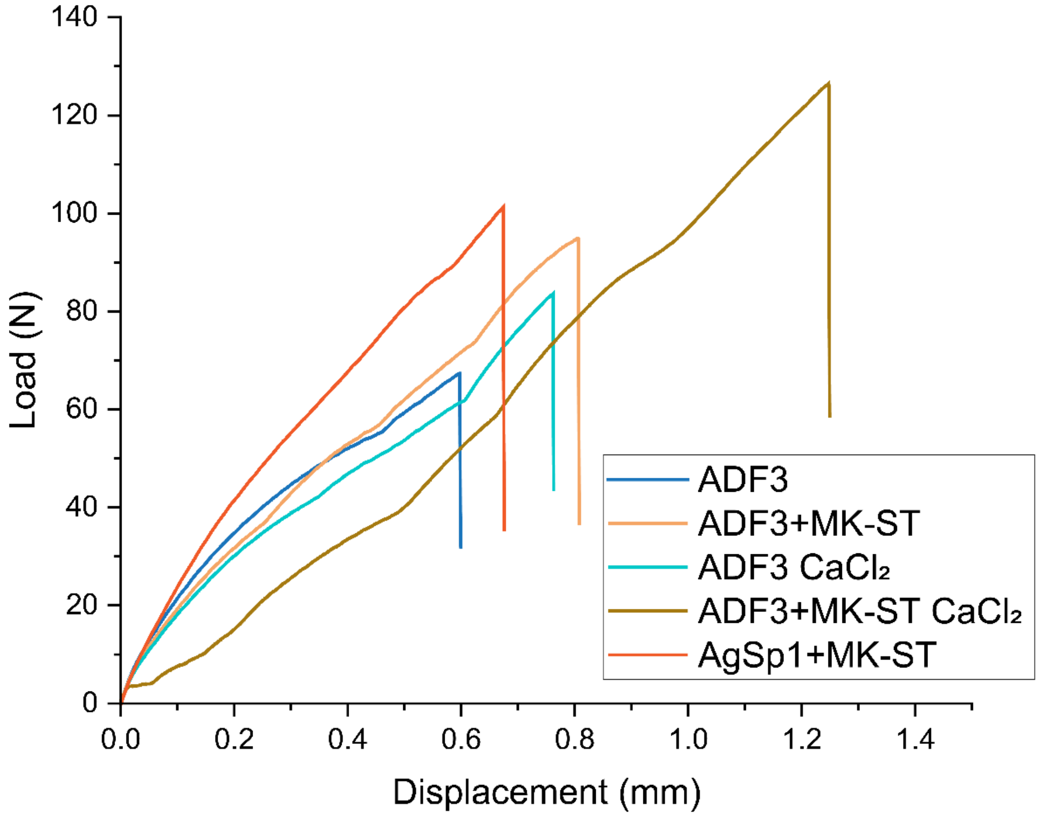

Figure S9. Representative load-displacement curves of measured silk samples.

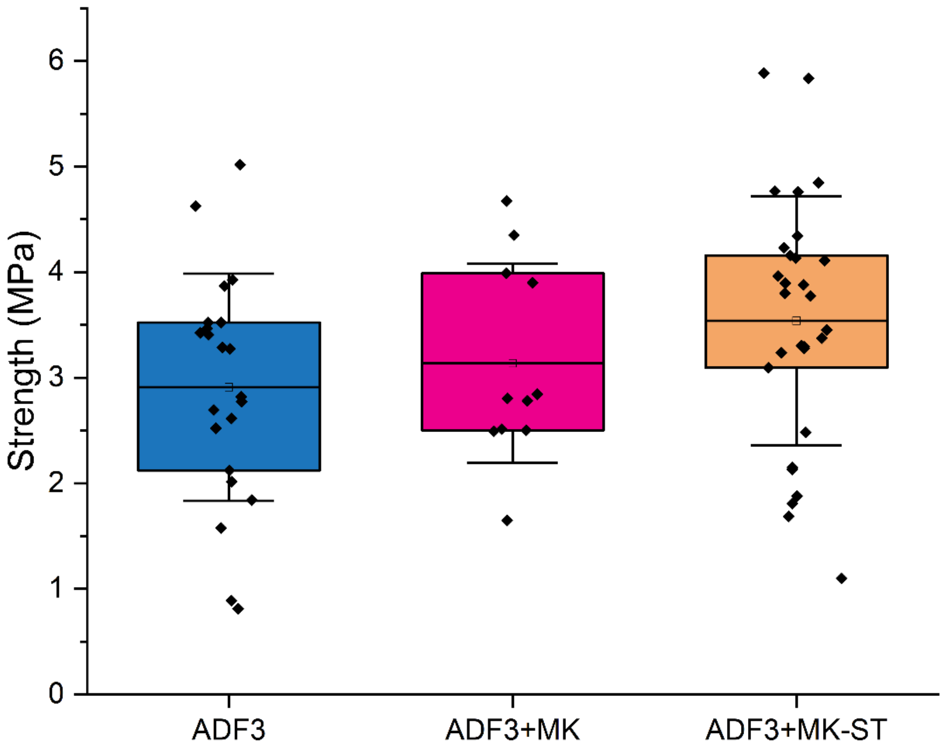

Figure S10. Adhesion strengths of ADF3 silk without phosphorylations (blue), with low level of modifications produced by co-expression with untagged kinase (pink), and with high level of phosphorylations obtained using the induced proximity approach (orange). The distribution of the individual measurements is shown. The mean is shown as a black line. Whiskers show standard deviation.

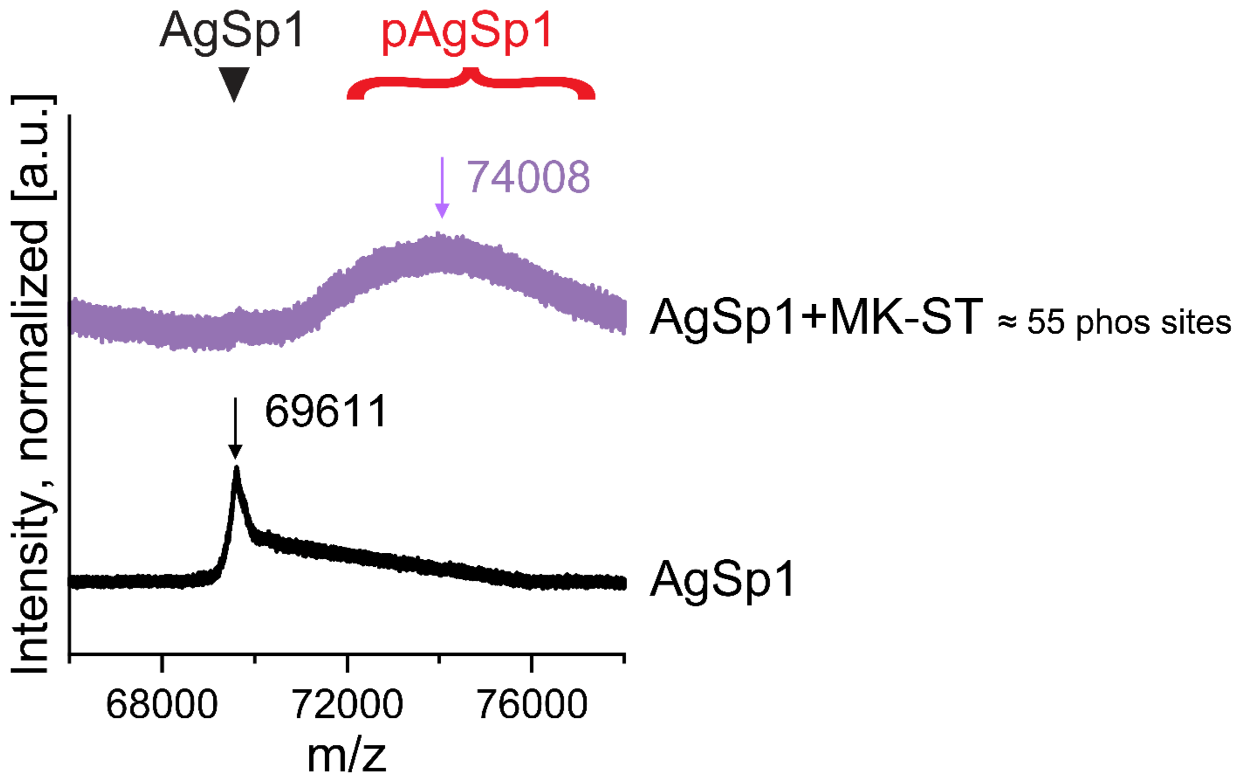

Figure S11. MALDI-TOF analysis of phosphorylated (violet) and unmodified (black) AgSp1 silk protein. Unmodified AgSp1 has a theoretical MW of 69466.71 Da without M1. Highest point of the phosphorylation peak has been evaluated by eye. Black arrow and red bracket indicate the expected apparent molecular weights of unmodified and phosphorylated AgSp1, respectively.

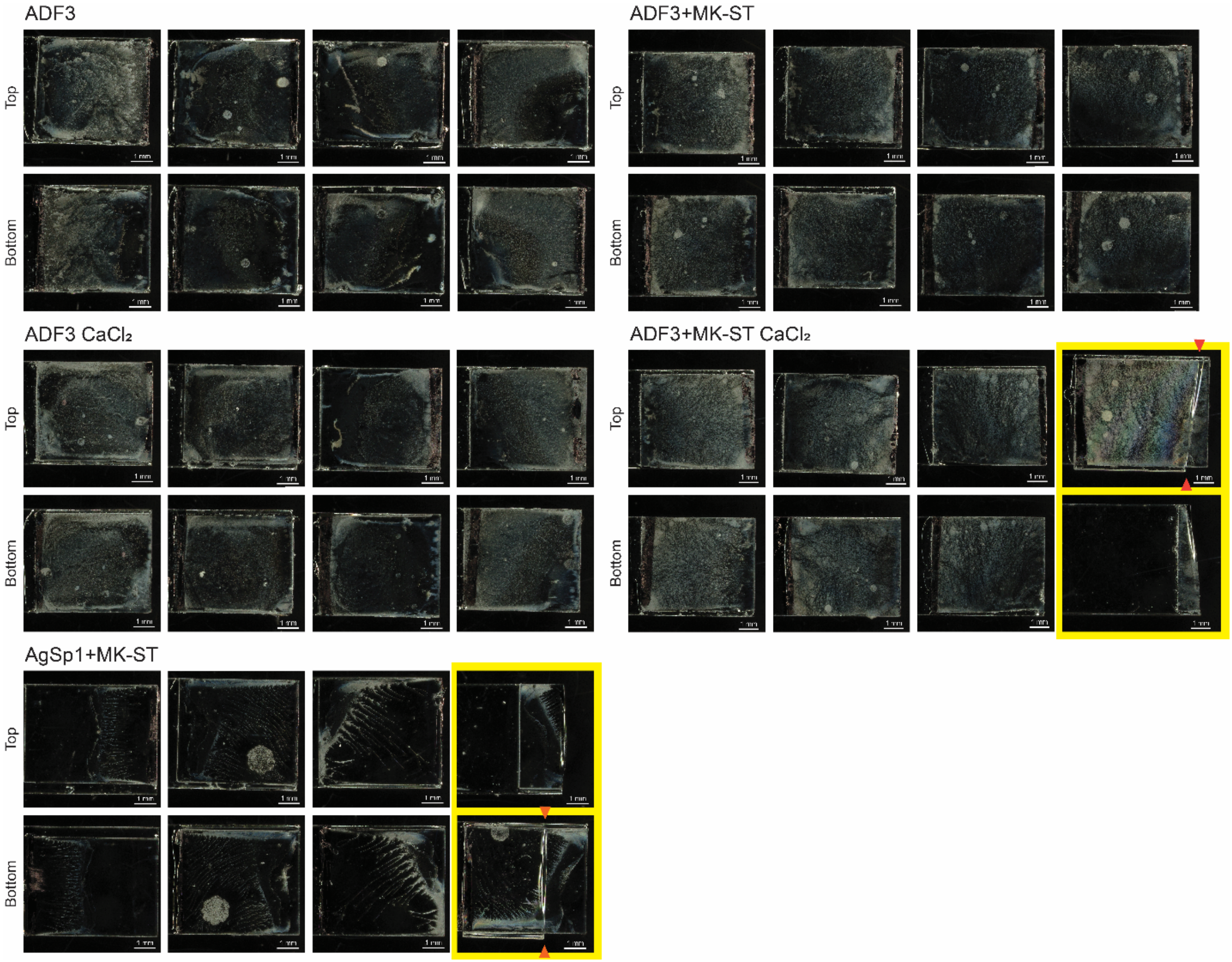

Figure S12. Representative light microscope images of the fractured surfaces.Examples of samples broken on the glass are shown in yellow and the edge of the broken glasses are indicated with orange arrows.

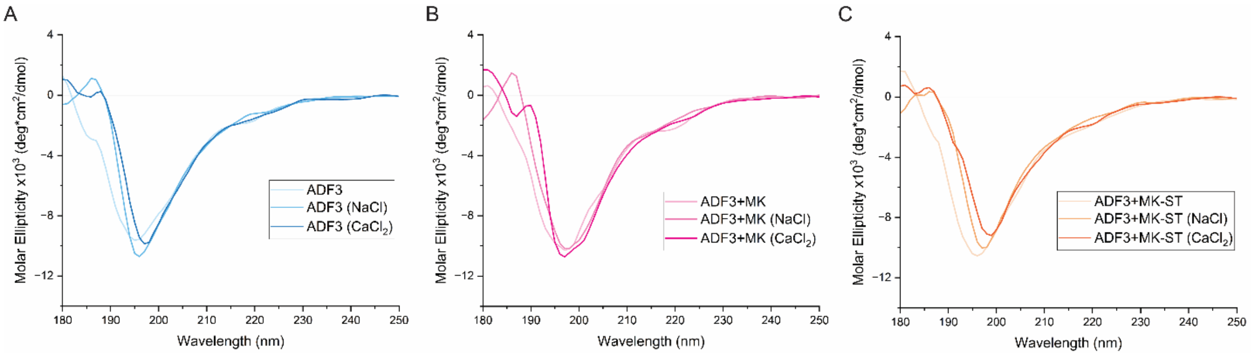

Figure S13. Analysis of the effect of phosphorylation and salts on the secondary structures of ADF3 silk protein. Circular dichroism spectra for ADF3 dragline silk without phosphorylations (A), with low level of modifications produced by co-expression with untagged kinase (B), and with high level of phosphorylations obtained using the induced proximity approach (C), with no salts, with 10 mM NaCl and with 10 mM $CaCl_2$.
